# Socioeconomic inequalities in food purchasing practices and expenditure patterns: Results from a cross-sectional household survey in western Kenya

**DOI:** 10.3389/fpubh.2023.943523

**Published:** 2023-01-26

**Authors:** Vincent Were, Louise Foley, Rosemary Musuva, Matthew Pearce, Pamela Wadende, Charles Lwanga, Ebele Mogo, Eleanor Turner-Moss, Charles Obonyo

**Affiliations:** ^1^Center for Global Health Research, Kenya Medical Research Institute, Kisumu, Kenya; ^2^Medical Research Council (MRC) Epidemiology Unit, University of Cambridge School of Clinical Medicine, Cambridge, United Kingdom; ^3^School of Education and Human Resource Development, Kisii University, Kisii, Kenya

**Keywords:** purchase, socioeconomic, inequalities, food, diet, consumption, expenditure, Kenya

## Abstract

**Introduction:**

Socioeconomic inequalities contribute to poor health. Inequitable access to diverse and healthy foods can be a risk factor for non-communicable diseases, especially in individuals of low socioeconomic status. We examined the extent of socioeconomic inequalities in food purchasing practices, expenditure, and consumption in a resource-poor setting in Kenya.

**Methods:**

We conducted a secondary analysis of baseline cross-sectional data from a natural experimental study with a sample size of 512 individuals from 376 households in western Kenya. Data were collected on household food sources, expenditure and food consumption. Household socioeconomic status (SES) was assessed using the multiple correspondence analysis (MCA) model. Concentration indices (Ci) and multivariable linear regression models were used to establish socioeconomic inequalities.

**Results:**

About half (47.9%) of individuals achieved a minimum level of dietary diversity with the majority coming from wealthier households. The two most consumed food groups were grains and roots (97.5%, *n* = 499) and dark green leafy vegetables (73.8%, *n* = 378), but these did not vary by SES. The consumption of dark green leafy vegetables was similar across wealth quantiles (Ci = 0.014, *p* = 0.314). Overall, the wealthier households spent significantly more money on food purchases with a median of USD 50 (IQR = 60) in a month compared to the poorest who spent a median of USD 40 (IQR = 40). Of all the sources of food, the highest amount was spent at open-air markets median of USD 20 (IQR = 30) and the expenditure did not vary significantly by SES (Ci = 0.4, *p* = 0.684). The higher the socioeconomic status the higher the total amount spent on food purchases. In multivariable regression analysis, household SES was a significant determinant of food expenditure [Adjusted coefficient = 6.09 (95%confidence interval CI = 2.19, 9.99)].

**Conclusion:**

Wealthier households spent more money on food compared to the poorest households, especially on buying food at supermarkets. Individuals from the poorest households were dominant in eating grains and roots and less likely to consume a variety of food groups, including pulses, dairy, eggs and fruits, and vegetables. Individuals from the poorest households were also less likely to achieve adequate dietary diversity. Deliberate policies on diet and nutrition are required to address socioeconomic inequalities in food purchasing practices.

## Introduction

Malnutrition and unhealthy diets are important risk factors for non-communicable diseases (1). International policies have focused on increasing the availability of inexpensive, high-calorie foods from staple cereal crops, which has reduced hunger for many. However, this has also affected food diversity and has displaced local, often healthier, diets (1). Access to diverse, micronutrient-rich foods such as fresh fruits, vegetables, legumes, pulses (beans, peas, and lentils), and nuts has not improved equally for everyone, and unhealthy foods with salt, sugars, saturated fats, and trans fats have become cheaper and more widely available (2). In Kenya, amongst the adult population, the prevalence of intake of processed foods high in salt was established to be 4.3% (95% confidence interval CI = 3.2–5.5%) and intake of intake of < 5 servings of fruits and/or vegetables daily was 94.0% (95% CI = 92.4–95.7%) while intake of processed food high in sugar was 1.6% (0.8–2.4%) (3). Overall, the proportion of Kenyans who always add salt to food before eating/while eating was established to be 23.6% (18.5–28.8%) in a survey conducted in 2015 (3). WHO's Commission on Social Determinants of Health defined health inequalities to be the result of the cumulative impact of decades of exposure to health risks of those who live in socioeconomically less advantaged circumstances (4).

A recent study established that food purchasers residing in low-income households with a low level of education are less likely to purchase foods high in fiber and low in sugar, fat, and salt (5). People who have a high income and a high degree of education are more likely to have access to resources for purchasing fruits, vegetables, and dairy products (6). According to a large-scale study conducted in India, both individual and environmental socioeconomic characteristics are linked to children's diverse dietary intake (7). Foods of lower nutritional value and lower quality are generally cheaper and were likely to be purchased by the majority of households of lower socioeconomic status (8).

Lower SES groups have shown significant associations with purchasing less healthy foods and drink while higher SES groups purchased high proportionally high-fat dairy and alcohol (9). Living in a higher socioeconomic affluent location is related to an inclination to consume healthful foods, according to a study conducted in Brisbane, Australia. However, the magnitude of the relationship was small (10). Children from wealthy families are assumed to grow better for a variety of reasons, one of which is that improved dietary adequacy may be one of the key ways that household wealth and resources translate into better outcomes for children. Wealthier households are likely to have more resources to purchase more food and so have more diverse diets than poorer households (11).

Other studies linked purchasing more fruits and vegetables and fewer unhealthy junk foods and beverages with the healthiness of one's choices (12). The frequency with which households frequented market-defined high-price and/or low-price supermarkets impacted their supermarket choice. The findings revealed that a higher occupational social class was connected with higher food expenditure, which was then linked to healthier purchasing (12). Individuals with the lowest quartile dietary costs, low education level and low income consumed fewer fruits and vegetables than individuals with higher quartile dietary costs, high education and high income (13).

Household size, composition, income, and education have depicted variation in food purchasing practices with a household with aging adults spending higher on vegetables and fruits whereas households with children spend greater on quantities of dairy products (14). Age and gender, on the other hand, are linked to the chance of using supermarket vouchers to buy healthy items (15).

In many nations, poor nutrition quality is a major public health concern. According to the most recent German diet report, poor food quality combined with a lack of exercise is to blame for the rising number of non-communicable diseases (16). Furthermore, poor diet quality is linked to the development of diet-related disorders such as diabetes, cardiovascular disease, and stroke (17). In Kenya, a study assessed the relationship between food security, health and wealth status. The results showed that the risk of stunting increased by 12% among children from food-insecure households and food security and wealth status jointly increased the risk of stunting (18).

Many developing nations are working to identify underlying gaps of inequalities in the population that will help inform policy formulation, which will drive interventions and minimization of risks among the populations. However, there is limited data on the relationship between individual or household social economic status (SES), food purchasing practices and expenditure patterns in resource-poor settings. This study, therefore, aimed to examine the extent of socioeconomic inequalities in food purchasing practices, expenditure and consumption in a resource-poor setting in western Kenya. The specific objectives were to:

1. To investigate the relationship between household SES and individual consumption of different food groups
2. To explore the distribution of households' expenditures on sources of food across household socioeconomic status
3. To investigate potential inequalities in achieved dietary diversity and the amount of money spent on various food retail.

## Materials and methods

### Study site and settings

Secondary data used in this analysis were from the Hypermarket, Foodscape and Health survey conducted in Kisumu and Homabay Counties in western Kenya (19). The hypermarket study was a mixed-methods natural experimental study that aimed to assess the potential impact of the investment in hypermarkets on food purchasing, dietary behaviors, and physical activity patterns of individuals living nearby. An area within a 2 KM radius of the new hypermarket in the Mamboleo area was selected as the intervention area and matched to the control area, Sofia in Homabay county. The intervention and control areas were matched by population density and geographical size. The land terrain, socioeconomic and food retail characteristics of both control and intervention areas were delineated as similar as possible (19). This secondary analysis has used baseline cross-sectional data from both the intervention and control areas as applied in a related analysis (20).

### Sample size and sampling procedure

The sample size for the study for this study has been described in the protocol (19). It was estimated that a minimum sample size of 300 (150 in each site) was adequate to detect a 5% difference in household food expenditure between the two sites (one site designated as intervention m Kisumu county, and the other as comparison in Homabay county, western Kenya), with 80% power and 95% confidence interval and 5% level of precision. The sampling frame was obtained from a list of households registered by community health volunteers (CHVs) in the area. As part of Kenya's health system structure, the CHVs are the first level of care and are mandated to register and maintain a household register for monthly follow-ups (21). We used this list which had about 2,000 households in each of the two sites. From the list, we asked CHVs to classify the households into three SES groups poor, middle or rich based on methods described by Foley et al. and Were et al. (19, 22). We also classified these households into three clusters based on the distance from a central predetermined landmark (2, 1.0, and 0.5 km) and further classified the households into four quadrants (NW, NE, SW, and SE). However, during the planning, we had anticipated higher attrition rates and, therefore, we aimed to recruit 50 households in each quadrant totaling 200 per site. We proportionately divided the 50 households between three levels of household SES (low, moderate, and high) and distance to a central landmark (2, 1, and 0.5 km). At the individual levels, we aimed at a maximum of five adults per household (19, 22). At the end of the survey, we achieved a higher number of 376 households in both sites (196 in Kisumu and 180 in Homabay). We also recruited 516 individuals in the survey (Kisumu 260 and 256 in Homabay).

### Data collection methods

The data were collected in March 2019 using CommCare, a mobile data collection tool (23). Household and individual questionnaires were administered by a team made up of one community health volunteer and two field workers. The household survey was completed by the household member in charge of food purchasing and the household head. The study considered a household head as an adult occupant of the household who is recognized and accepted as the head by other household members. The household survey had questions on household composition and income and household food purchasing. The individual survey included a recall of food consumed in the previous 24 h.

### Main exposure variable

#### Household socioeconomic status

The socioeconomic status of households was established using variables that differentiated households into wealth quintiles. These included ownership of durable assets, household characteristics and utilities [dwelling type; sources of water (piped water, public tap, dug well, rainwater collection, vendors, and surface water)]; availability of electricity; cooking appliance (stone fire, Jiko, kerosene stove, and gas cooker); source of energy for cooking (firewood, electricity, liquified petroleum, biogas, kerosene, and charcoal); rearing of an animal; growing of food; availability of refrigerator; and ownership of a private car). A composite index was then created and classified into five wealth quintiles using the Multiple Corresponding Analysis (MCA) (24), an improvement of the traditional principal component analysis (PCA) model (25). The first quintile was labeled as the poorest, and the fifth quintile as the least poor (wealthiest). The household heads reported the ownership of the assets and utilities of the households as was asked by the research assistants (22). The ownership of assets and utilities was self, or proxy-reported. The responses on ownership of assets were coded as binary no/yes variables.

### Study outcomes

#### Household food expenditure

Household food expenditure was assessed by asking the food purchaser or a proxy the amount of money in Kenya shillings (KSH) spent on food in different food outlets in the last month. The food purchasers were also asked about the total amount spent on food consumed in the household in the last month. The amount of money spent on food was quoted in Kenyan shillings and converted to US dollars using an exchange rate of 1 USD to KES 100.

#### Household food retail sources

This study defines food retail source as the origin(s) from which food consumed in the household was acquired 1 month before the study. The study divided food sources into two categories: food retail sources and food sources. Food sources included retail and non-retail methods of acquiring food. The food retail category included food retail outlets from which households bought food (Table 1). The household member responsible for food purchase reported all food retail sources from which the household acquire food. Each food retail source was coded as a binary no/yes variable.

**Table 1:** Description of food retail sources included in the study.

**Food retail source**	**Description**
• Supermarket (yes/no)	• A large store selling household items and a variety of food
• Open-air market (yes/no)	• A public market where local vendors sell merchandise and food
• Kiosk (yes/no)	• A small open-front structure selling a small range of food and other goods
• General shop (yes/no)	• A small local shop selling commonly used goods
• Specialized shop (yes/no)	• A small store selling a specific category of goods
• Informal (roadside) vendor (yes/no)	• Street vendors without a fixed permanent structure
• Restaurant (yes/no)	• A place where people eat prepared meals
• Fast food (yes/no)	• A place where people get quickly prepared processed meals
• Café (yes/no)	• A place where people order cold and hot beverages and take light meals
• Online (yes/no)	• A website selling unprepared food over the internet and can have them delivered to customers' homes or nearby towns
• Other	• Other food retail outlets used by households

### An individual's diet diversity

An uninterrupted multiple-pass pen and paper 24-h recall diet was done among adults who took part in the survey (26). The research assistants coded each reported drink or food to the appropriate food group on the electronic data collection tool with guidance from a food list. The study then used the Minimum Dietary Diversity for Women (MDD-W) indicator from the Food and Agriculture Organization of the United Nations to assess individual dietary diversity (27). Drinks and food consumed by adult individuals in the households were classified by MDD-W into 10 food groups (grains and roots; pulses; nuts and seeds; dairy; meat, poultry, fish; eggs; dark green leafy vegetables; vitamin A-rich vegetables and fruits; other vegetables; other fruits). Achievement of minimum dietary diversity was coded as a binary no/yes variable with minimum dietary diversity implying a dietary diversity score of 5 and above. This approach has been used in recent similar studies and a previous analysis using this dataset (20, 28).

### Covariates

The analysis included adjusted linear regression modeling to establish the potential relationship between socioeconomic status and total expenditure on food. The variables included sociodemographic characteristics of the households' heads (age in years), gender (male or female), the highest level of education (primary or less, secondary, post-secondary), working status (unemployed, employed/retired), years lived in the local area and household size. We first conducted a bivariable linear regression analysis to establish an association between household SES and food expenditure. We established a significant association. We then included prior-identified covariates known to influence household expenditure based on literature (sex, age, highest level of education, and working status), years lived in dwelling, and household size) and added them to the model as a block (enter method). It was done using Stata software version 15 and the approach was an enter selection method. After establishing a significant association between SES and household expenditure in step 1, a block of covariates was entered in the second step.

### Data management and analysis

The study used STATA (STATA 15.0; Stata Corp, College Station, TX), a statistical software, to perform data management and statistical analyses. Statistical test results with a probability value (*p*-value) < 0.05 were considered significant (95% confidence level). Sampling weights were assigned to variables before adjusting for clustering at the household level in the multivariable regression models. Weights were calculated by dividing the targeted sample size (sample distribution of households classified by community health volunteers) from each stratum by the actual sample size obtained by field workers during data collection. The population was stratified by geographical location (NE, NW, SE, and SW) and socioeconomic status (low SES, middle SES, and high SES). Consistency checks and validation were conducted on the household and individual datasets to identify and correct errors before they were merged using household identifiers.

### Descriptive statistics

Descriptive statistics such as frequency distribution and proportions were used to describe the distribution of individuals' sociodemographic characteristics, households' characteristics, and food groups consumed during 24-h dietary recall by household wealth quintiles. All the continuous variables were assessed for normality using probability plots and the Shapiro-Francia *W'*-test. Skewed data were analyzed using a median and interquartile range.

### Dietary diversity consumed food groups and household food expenditure by household socioeconomic status

Households were classified as poorest, second poor, middle poor, less poor, and least poor. We assessed socioeconomic health inequality using a concentration index. The concentration index is a relative measure of inequality that indicates the extent to which a health indicator is concentrated among the disadvantaged or the advantaged (29, 30). Given that a population is ranked by increasing socioeconomic status, the concentration index has a negative value when the health indicator is concentrated among the disadvantaged and a positive value when the health indicator is concentrated among the advantaged (29, 30). If a single individual (the smallest possible population subgroup) accounted for 100% of a health indicator in a population (the highest relative inequality that is theoretically possible), this would cause the concentration index to approach its maximum absolute value of either −1 or +1. Where the 95% CI for the Ci estimate does not include zero or where the *p*-value was < 0.05, the results were considered statistically significant.

### Households' expenditure on different types of food retail by household socioeconomic status

To assess the variation in the amount spent on different types of food retail by household socioeconomic status, the study used concentration indices to assess the inequality in the absolute amount spent on sources of food across different households' wealth quintiles (unadjusted for covariates). We considered additionally exploring expenditure relative to household income, however, we decided against this because of the problems with reporting this data in low-income households described above. We also assessed the percentage of total food expenditure for each SES to assess potential catastrophic expenditures.

A generalized linear regression model with an identity link function and a Gaussian family was used to assess the linear relationship between socioeconomic status and the total amount spent on food from food outlets by the households. The model was adjusted for covariates, including the characteristics of the households including households' heads' sex, age, the highest level of education, working status, years lived in the dwelling, and household size.

## Results

### Characteristics of the households in the study

Descriptive statistics of the household characteristics by socioeconomic status are presented in Table 2. Of the 400 households contacted, 376 (94%) completed the survey.

**Table 2:** Characteristics of the households by socioeconomic status (*n* = 376).

**Variables**	**Wealth quintile**					**Least poor**
	**Overall**	**Poorest**	**2**	**3**	**4**	***n*** **(%)**
	***n*** **(%)**	***n*** **(%)**	***n*** **(%)**	***n*** **(%)**	***n*** **(%)**	
**Male household head (*****n*** **=** **369)**	188 (50.9)	44 (52.6)	37 (56.2)	43 (58.6)	33 (49.8)	30 (38.2)
**Age of the household in years (*****n*** **=** **368) Median (IQR)**	40 (23.0)	44 (25.1)	47.5 (26.0)	42 (22.0)	37 (20.3)	33 (17.0)
**Highest education level of household head (*****n*** = **369)**						
Primary or less	202 (54.8)	67 (81.1)	48 (72.5)	24 (32.8)	27 (40.8)	35 (44.8)
Secondary	93 (25.2)	13 (15.6)	10 (14.8)	25 (33.5)	20 (30)	25 (32.1)
Post-secondary	74 (20.0)	3 (3.3)	8 (12.7)	25 (33.7)	20 (29.2)	18 (23.1)
**Working status of household head (*****n*** = **369)**						
Unemployed	119 (32.3)	46 (55.0)	29 (43.3)	8 (11.4)	16 (24.2)	20 (25.6)
Employed/retired	250 (67.7)	37 (45.0)	37 (56.7)	66 (88.6)	51 (75.8)	59 (74.4)
**Household size (*****n*** **=** **376), median (IQR)**	4 (3.0)	4 (3.0)	4 (3.0)	4 (3.0)	3 (3.0)	3 (2.0)
**Years living in the local area (*****n*** **=** **376), median (IQR)**	12 (23.0)	18 (23.0)	17 (32.0)	16 (28.3)	7 (16.0)	4 (16.0)
**Dwelling type (*****n*** = **376)**						
Bungalow	49 (12.9)	7 (7.7)	10 (14.2)	17 (22.5)	11 (16.6)	4 (5.5)
Flat	47 (12.5)	7 (8.2)	2 (3.7)	10 (13.2)	10 (14.2)	18 (22.1)
Maisonette	18 (4.8)	0 (0)	2 (2.6)	9 (12.3)	2 (3.4)	5 (5.8)
Swahili	17 (4.6)	4 (5.1)	6 (8.4)	6 (7.6)	2 (2.5)	0 (0)
Shanti	145 (38.6)	10 (12.4)	34 (51.4)	27 (35.7)	27 (38.7)	47 (57.7)
Manyatta/traditional house	60 (16.0)	50 (58.8)	3 (5.1)	1 (1.2)	6 (9.0)	0 (0)
Other	40 (10.7)	7 (7.9)	10 (14.6)	6 (7.5)	11 (15.7)	7 (8.9)
**Household has electricity (*****n*** **=** **376)**	237 (63.0)	17 (20.1)	43 (63.9)	60 (81.0)	51 (74.0)	66 (81.3)
**Main water source (*****n*** = **376)**						
Piped water	100 (26.6)	13 (15.4)	10 (15.0)	33 (44.5)	23 (32.7)	21 (26.4)
Public tap/standpipe	147 (39.1)	9 (10.5)	30 (45.0)	23 (31.5)	32 (46.0)	53 (65.4)
Well	24 (6.4)	15 (17.4)	2 (2.5)	3 (3.6)	3 (5.0)	2 (2.1)
Vendors	29 (7.7)	4 (4.6)	3 (3.8)	6 (8.2)	11 (16.4)	5 (6.1)
Surface water	66 (17.5)	43 (51.2)	18 (27.1)	4 (5.7)	0 (0)	0 (0)
Other	10 (2.7)	1 (0.9)	4 (6.6)	5 (6.4)	0 (0)	0 (0)
**Own refrigerator (*****n*** **=** **376)**	60 (15.9)	1 (1.3)	5 (7.6)	28 (37.1)	14 (19.7)	13 (15.6)
**own car (*****n*** **=** **376)**	37 (9.8)	0 (0)	4 (6.3)	24 (31.7)	5 (7.7)	4 (4.6)

Household size: the number of individuals currently living in the households.

On average, the household members had stayed in the households for 12 years (Interquartile range IQR = 23), with the poorer (median = 18 years, IQR = 23) staying longer than the richer (median = 4 years, IQR = 16) in the survey area. Poorer households had many inhabitants (median = 4, IQR = 3) compared to richer (median = 3, IQR = 2) households, and a majority of male-headed households were poorer. Only three (3.3%) household heads from poorer households and 18 (23.1%) household heads from richer households had attained post-secondary education. Household heads who were employed or retired were mostly found in richer households (*n* = 59, 74.4%) compared to poorer households (*n* = 37, 45%). The Shanti houses were the most common (*n* = 145, 38.6%) dwelling type in the survey area, and most (*n* = 47, 57.7%) of them were categorized under the wealthier quintile. Electricity, refrigerator, and private car were mostly found in richer households. Public tap is the main water source for most of the households in the survey area and is mostly accessed by richer households (Table 2).

### Characteristics of the study participants by wealth quintile (*n* = 512)

A total of 512 individuals were included in the survey. The distribution of characteristics of study participants by wealth quintile is presented in Table 1. Females were 70.8 % (*n* = 362) and males were 29.2% (*n* = 150). The participant's ages were categorized into 18–34 years (49.6%; *n* = 254), 35–52 years (31.4%; *n* = 161), and>52 years (19%; *n* = 97). The majority of individuals in the poorest quintile had normal body mass index (BMI), 47.1% (*n* = 241) and only 19.2% (*n* = 99) were obese. On the other hand, among the least poor, 43.8 % (*n* = 47) were normal and the minority were under-weight 6.4% (*n* = 7). On chronic diseases, the prevalence of self-reported diabetes was 1.6 % (*n* = 8), stroke 1.7% (*n* = 9), and high blood pressure 16.3% (*n* = 83). Of those reporting high blood pressure, 11.1% (*n* = 12) were from the least poor households and 18.8% (*n* = 22) were from the poorest households (Table 3).

**Table 3:** Descriptive statistics of individual participants by household wealth quintiles in hypermarket study, Western Region, Kenya (*n* = 512).

**Variables**	**Wealth quintile**					
	**Total**	**Poorest**	**2nd poor**	**3rd poor**	**4th least poor**	**Wealthiest**
	***n*** **(%)**	***n*** **(%)**	***n*** **(%)**	***n*** **(%)**	***n*** **(%)**	***n*** **(%)**
**Gender**						
Male	150 (29.2)	34 (29.1)	34 (36.8)	34 (32.4)	28 (30.1)	20 (18.8)
Female	362 (70.8)	82 (70.9)	59 (63.2)	70 (67.6)	65 (69.9)	86 (81.2)
**Age**						
18–34 (years)	254 (49.6)	57 (49.4)	30 (31.8)	46 (44.1)	59 (63.6)	62 (58.4)
35–52	161 (31.4)	34 (29.7)	35 (37.1)	30 (29.1)	23 (24.5)	39 (36.5)
53 and above	97 (19)	24 (20.9)	29 (31.1)	28 (26.8)	11 (11.9)	5 (5.1)
**BMI (kg/m** ^2^ **)**						
Underweight (< 18.5 kg/m^2^)	47 (9.1)	15 (12.6)	11 (12.2)	9 (8.5)	5 (5.6)	7 (6.4)
Normal (18.5–24.9 kg/m^2^)	241 (47.1)	63 (54.6)	46 (49.5)	47 (45.7)	38 (40.7)	47 (43.8)
Overweight (25–29.9 m^2^)	126 (24.6)	15 (13.4)	17 (18.7)	30 (29.3)	29 (31.4)	33 (31.2)
Obese (≥30 kg/m^2^)	99 (19.2)	23 (19.5)	18 (19.6)	17 (16.6)	21 (22.3)	20 (18.6)
**Achieved diet diversity**						
No	267 (52.1)	81 (70.2)	62 (66.8)	40 (38.4)	39 (41.4)	45 (42.6)
Yes	245 (47.9)	34 (29.8)	31 (33.2)	64 (61.7)	55 (58.6)	61 (57.4)
**Chronic diseases**						
Diabetes	8 (1.6)	2 (1.7)	3 (2.7)	1 (0.8)	2 (1.8)	1 (1.1)
Stroke	9 (1.7)	2 (1.4)	2 (1.8)	2 (1.6)	2 (1.9)	2 (1.9)
High blood pressure	83 (16.3)	22 (18.8)	18 (18.9)	21 (20.2)	11 (12)	12 (11.1)

### Relationship of household socioeconomic status with dietary diversity and food groups consumed

Of the survey participants, 47.9% (*n* = 245) achieved diet diversity based on foods consumed, and out of these, 29.8% (*n* = 34) were from the poorest households and 57.4% were from the least poor households. “Grains and roots” were the most reported food group, with 97.5% of individuals reporting consumption from this food group on the previous day. Consumption of grains and roots was not significantly different across socioeconomic status (*p*-value = 0.236). By contrast, the consumption of pulses was 26.8% was significantly concentrated amongst wealthier individuals (Ci = 0.041, *p*-value = 0.018). The consumption of dark green leafy vegetables was similar across wealth quantiles (Ci = 0.014, *p*-value = 0.314). The least common food group consumed was nuts and seeds 7.6% (*n* = 39) and the level of consumption was similar across socioeconomic groups (*p*-value = 0.270). The result also indicated a significant inequality in the consumption of pulses, dairy foods, eggs, vitamin A-rich vegetables and fruits, and other fruits across different wealth quintiles. The concentration indices indicate that pulses (CI = 0.041, *p*-value = 0.018), dairy products (CI = 0.046, *p*-value = 0.012), eggs (CI = 0.027, *p*-value = 0.002), vitamin A-rich vegetables and fruits (CI = 0.061, *p*-value < 0.001), and other fruits (CI = 0.025, *p*-value = 0.027) were mostly consumed by the least poor compared to the poorer (Table 4).

**Table 4:** The proportion of types of foods consumed among individuals across household socioeconomic status in a hypermarket study in Western Region, Kenya (*n* = 512).

**Foods consumed**		**Wealth quintiles**					**Concentration index (Ci)**		
	**Total**	**Poorest**	**2nd poorer**	**3rd middle poor**	**4th less wealthy**	**Wealthiest**	**CI**	**Std error**	***p*** **-value**
	***n*** **(%)**	***n*** **(%)**	***n*** **(%)**	***n*** **(%)**	***n*** **(%)**	***n*** **(%)**			
Grains and roots	499 (97.5)	100 (97.1)	101 (99)	106 (99.1)	96 (97)	96 (95.1)	−0.005	0.004	0.236
Pulses	137 (26.8)	13 (12.6)	18 (17.7)	36 (33.6)	29 (29.3)	41 (40.6)	0.041^*^	0.017	**0.018**
Nuts and seeds	39 (7.6)	5 (4.9)	7 (6.9)	9 (8.4)	12 (12.1)	6 (5.9)	0.007	0.006	0.270
Dairy	294 (57.4)	45 (43.7)	49 (48)	79 (73.8)	61 (61.6)	60 (59.4)	0.046^*^	0.018	**0.012**
Meat, Poultry, Fish	297 (58)	58 (56.3)	62 (60.8)	68 (63.6)	54 (54.6)	55 (54.5)	−0.003	0.019	0.874
Eggs	64 (12.5)	5 (4.9)	5 (4.9)	25 (23.4)	16 (16.2)	13 (12.9)	0.027^*^	0.009	**0.002**
Dark green leafy vegetables	378 (73.8)	70 (68)	70 (68.6)	83 (77.6)	79 (79.8)	76 (75.3)	0.014	0.014	0.314
Vitamin A-rich vegetables and fruits	149 (29.1)	12 (11.7)	15 (14.7)	43 (40.2)	38 (38.4)	41 (40.6)	0.061^*^	0.013	**< 0.001**
Other vegetables	277 (54.1)	56 (54.4)	52 (51)	61 (57)	51 (51.5)	57 (56.4)	0.022	0.018	0.238
Other fruits	100 (19.5)	8 (7.8)	20 (19.6)	23 (21.5)	22 (22.2)	27 (26.7)	0.025^*^	0.011	**0.027**

Ci, Concentration index.

Negative values show dominance by the poorest and positive values show dominance by the less poor. Bold values indicate the statistically significant (*p*-values < 0.05).

### Relationship of household socioeconomic status with expenditure at different types of food retail

Overall, the wealthier households spent significantly more money on food purchases median of USD 50 (IQR = 60) in the last month compared to the poorest who spent a median of USD 40 (IQR = 40). The amount spent on food from a supermarket was the highest median USD 20 (IQR = 30). The inequality in the amount spent on food at different food retail is presented in Table 5. A significant inequality in the total amount spent on food was observed, where the wealthier households spent more money on food compared to the poorest households (CI = 5.28, *p*-value = 0.002). The richer households also spent more money on food from supermarkets (CI = 3.3, *p*-value < 0.001) and specialized shops (CI = 0.2, *p*-value = 0.021) compared to the poorest households (Table 5).

**Table 5:** The median amount spent on food by households and inequality in the amount spent on food across different socioeconomic status in Western Region, Kenya (*n* = 376).

**Variables**	**Wealth quintile**						**Concentration index**		
	**Total**	**Poorest**	**2nd poor**	**3rd poor**	**4th less**	**Least poor**	**CI**	**Std. error**	***p*** **-value**
	**Median (IQR)**	**Median (IQR)**	**Median (IQR)**	**Median (IQR)**	**Median (IQR)**	**Median (IQR)**			
Household expenditure on food (in USD)	50 (60)	40 (40)	40 (70)	70 (71)	70 (60)	50 (60)	5.28^*^	1.66	**0.002**
**Expenditure on sources of food (in USD)**									
Supermarket (*n* = 212/376)	5 (20)	0 (5)	0 (15)	20 (33)	15 (40)	10 (20)	3.3^*^	0.67	**< 0.001**
Open air market (*n* = 365/376)	20 (30)	20 (20)	20 (30)	25 (30)	25 (40)	20 (20)	0.4	0.90	0.684
Kiosk (309/376	5 (13)	5 (8)	5 (14)	10 (10)	10 (18)	5 (5)	0.6	0.36	0.080
General shop (*n* = 168/376)	0 (9)	0 (6)	0 (5)	0 (10)	2 (10)	0 (7)	0.3	0.34	0.426
Specialized shop (*n* = 61/376)	0 (0)	0 (0)	0 (0)	0 (0)	0 (0)	0 (0)	0.2^*^	0.08	**0.021**
Informal (roadside) vendor (*n* = 112/376)	0 (2)	0 (0)	0 (1)	0 (0)	0 (5)	0 (5)	0.4	0.20	0.065
Restaurant (*n* = 112/376)	0 (0)	0 (0)	0 (0)	0 (0)	0 (0)	0 (0)	0.0	0.00	0.065
Fast foods (*n* = 14/376)	0 (0)	0 (0)	0 (0)	0 (0)	0 (0)	0 (0)	0.0	0.03	0.605
Café (*n* = 4/376)	0 (0)	0 (0)	0 (0)	0 (0)	0 (0)	0 (0)	0.0	0.02	0.286
Online (*n* = 19/376)	0 (0)	0 (0)	0 (0)	0 (0)	0 (0)	0 (0)	0.0	0.05	0.726

IQR, Interquartile range; Ci, Concentration index; std, standard.

Bolded, *p*-value < 0.05 shows a significant test result.

### Average amount and proportion spent in food outlets by socioeconomic status

Table 6 presents the median amount of money spent on food from different food outlets and the proportion of the total amount spent at each food outlet by socioeconomic status. Of the total amount spent on food in the last month before the survey, a bigger proportion was spent by the least poor households (19.9%). The result indicated that the money spent on food from the open-air market was higher than the money spent on food from other food outlets. A larger proportion of the money spent on food from the open-air market was spent by households in the 4th wealth quintile (22.2%).

**Table 6:** Median monthly expenditure and proportion of expenditures in food outlets stratified by socioeconomic status in hypermarket study, Western Region, Kenya (*n* = 376).

**Food outlets**	***n* (%)**	**Total Kshs (%)**	**Poorest Kshs (%)**	**2nd poor Kshs (%)**	**3rd poor Kshs (%)**	**4th less poor Ksh (%)**	**Least poor Kshs (%)**
Money spent on food	376 (100.0)	3,196.98 (50)	40 (14.2)	40 (17.3)	70 (25.1)	70 (23.6)	50 (19.9)
**Expenditure on sources of food (in USD)**							
Supermarket	212 (56.4)	795.83 (5)	0 (4.8)	0 (9.7)	20 (40)	15 (25.6)	10 (20)
Open-air market	365 (97.1)	1,356.96 (20)	20 (18.9)	20 (19.8)	25 (20.5)	25 (22.6)	20 (18.3)
Kiosk	309 (82.2)	480.76 (5)	5 (16.7)	5 (18.5)	10 (20.2)	10 (23.4)	5 (21.2)
General shop	168 (44.7)	262.68 (0)	0 (19.2)	0 (17.7)	0 (17.9)	2 (23.8)	0 (21.4)
Specialized shop	61 (16.2)	49.79 (0)	0 (6.3)	0 (14.5)	0 (29.7)	0 (30.9)	0 (18.7)
Informal shop	112 (29.8)	107.16 (0)	0 (12.3)	0 (21)	0 (13.2)	0 (23.1)	0 (30.3)
Restaurant	112 (29.8)	1.07 (0)	0 (12.3)	0 (21)	0 (13.2)	0 (23.1)	0 (30.3)
Fast food	14 (3.7)	10.77 (0)	0 (13)	0 (21.1)	0 (36.3)	0 (29.7)	0 (0)
Café	4 (1.1)	4.08 (0)	0 (25.6)	0 (49.3)	0 (0)	0 (25.1)	0 (0)
Online	19 (5.1)	13.92 (0)	0 (0)	0 (50.4)	0 (9.9)	0 (16.9)	0 (22.8)

### Socioeconomic inequalities in achieved dietary diversity

**Figure 1:**
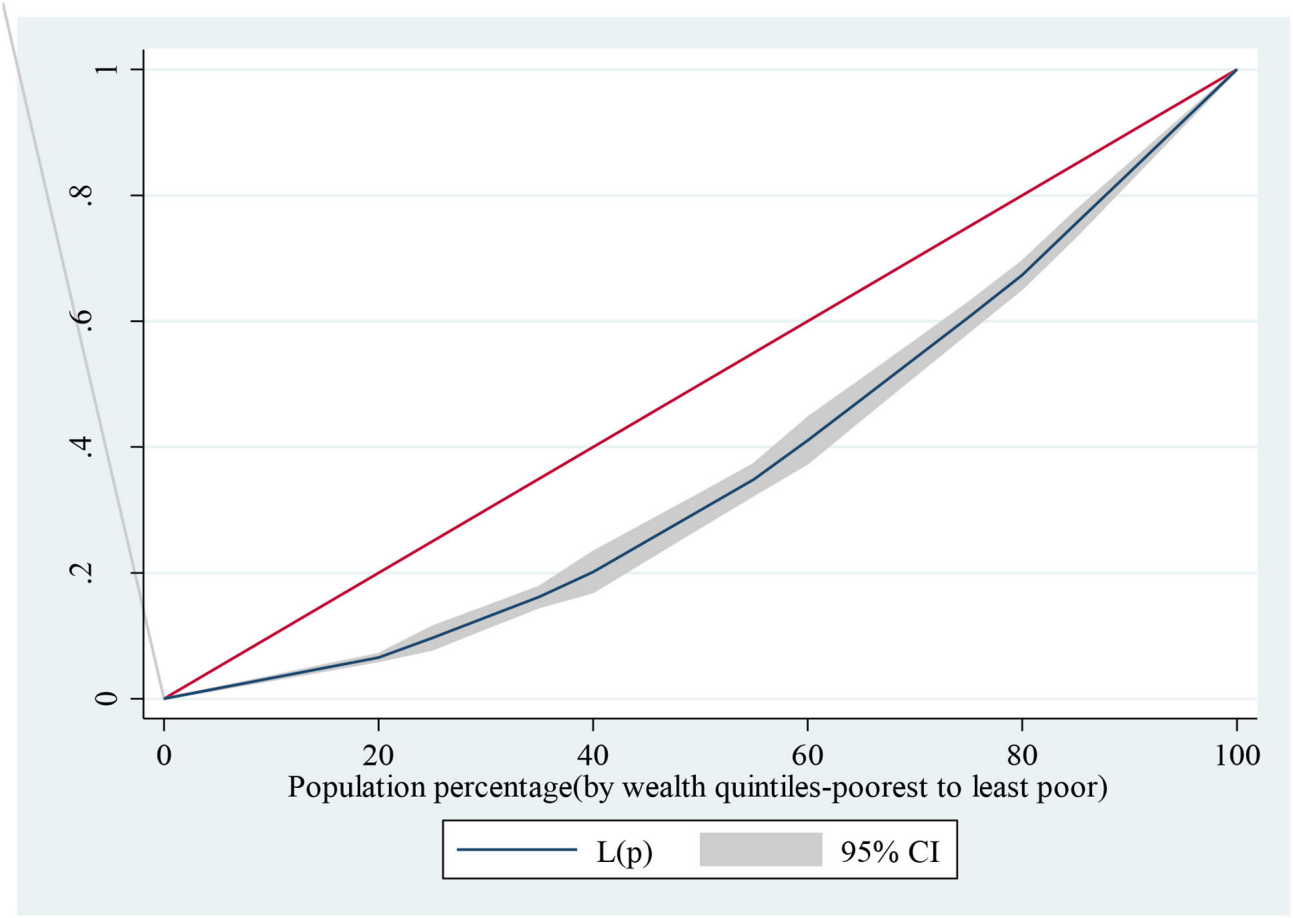
Wealth inequality among individuals who achieved dietary diversity.

Figure 1 shows the results of the Lorenz curve to establish the extent of socioeconomic inequalities in achieving dietary diversity in foods consumed. The result shows that dietary diversity was dominated by the less poor individuals as the inequality curves fall below the line of equality.

### Inequality of amount of money used to purchase food in various outlets stratified by household socioeconomic status

**Figure 2:**
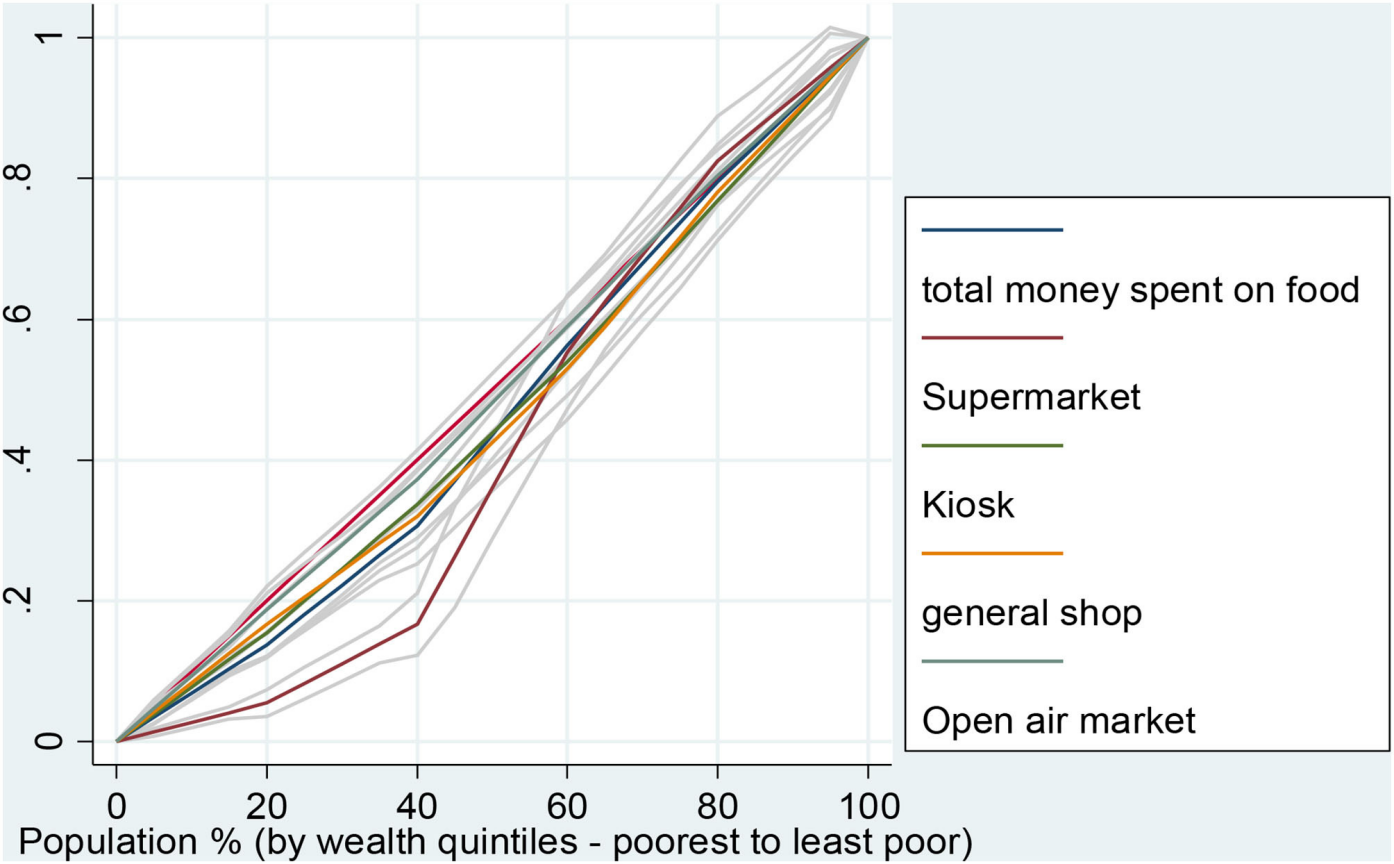
Inequality of the amount of money used to purchase food in various outlets by household wealth quintiles.

The study assessed the inequality in the amount spent in different food outlets by wealth quintiles using the Lorenz curve. Figure 2 shows that the money spent in supermarkets was dominated by the less poor and this was significant. It also shows that less poor households dominated spending more money on kiosks, open-air markets, and general shops.

### Association between households' socioeconomic status and expenditure on food

Table 7 presents the linear regression marginal effects of household socioeconomic status on the total amount spent on food by households. In the unadjusted linear regression model, socioeconomic status had a positive and significant relationship with the total amount spent on food by the households (Unadjusted coefficient = 6.42, CI: 2.47, 10.37). After adjusting for covariates (sex, age, highest level of education, and working status), years lived in dwelling, and household size, socioeconomic status still showed a positive relationship with the total amount of money spent on food by the households (Adjusted coefficient = 6.09, CI: 2.19, 9.99).

**Table 7:** Association of household socioeconomic status with expenditure on food in Western Region, Kenya.

	**Outcome: Total amount spent on food by households**	
	**Unadjusted coefficient**	**Adjusted coefficient**
	**(95% confidence interval)**	**(95% confidence interval)**
Socioeconomic status (poorest-1 to least poor - 5)	6.42 (2.47,10.37)^*^	6.09 (2.19, 9.99)^*^

* Significant at 0.05 level, unadjusted linear regression model: unadjusted coefficient, n = 376, adjusted linear regression model: adjusted for household head characteristics (sex, age, highest level of education, and working status), years lived in dwelling, and household size, n = 368 (eight households had missing data on the head of the household.

## Discussion

We aimed to examine the extent of socioeconomic inequalities in food consumption, expenditure, and dietary diversity in a resource-poor setting in western Kenya. We first assessed the achievement of dietary diversity by social-economic status and the results showed that nearly half of all participants had achieved dietary diversity and were dominated by individuals from less poor households. Grains and roots were the most consumed food groups and the consumption level was not different by household SES. The result showed that there was significant inequality in the consumption of pulses, dairy products, eggs, vitamin A-rich vegetables and fruits among individuals coming from different socioeconomic backgrounds. Pulses, dairy products, eggs, vitamin A-rich vegetables, and fruits were mostly consumed by the less poor compared to the poorest. These results are comparable to those of a study in Algeria which concluded that the most consumed food group among adult men and women were the staple foods followed by flesh food, and dairy, while the least consumed food group was Vitamin A-rich dark green leafy vegetables followed by nuts and seeds (31). Similarly, our study found that the most consumed food group was grains and roots, while the least consumed food group was nuts and seeds. Additionally, our study also established that pulses, dairy products, eggs, vitamin A-rich vegetables, and fruits were mostly consumed by individuals from less poor households.

According to World Health Organization (WHO), most of the risk factors for NCDs are preventable and have identified four key factors which require interventions. These include tobacco use, physical inactivity, unhealthy diet, and the harmful use of alcohol) that lead to four key metabolic/physiological changes (raised blood pressure, overweight/obesity, raised blood glucose, and raised cholesterol) (32). Our study has established that there exist socioeconomic inequalities in the consumption, purchasing and expenditure of various sources of foods which will imply that interventions should be targeted to individuals in each socioeconomic group. Wealthier households are known to have higher purchasing power and can assess almost types of foods; however, the consumption and expenditure patterns still expose them to the risk of these NCDs. Similarly, individuals in lower SES already are at risk of NCDs given that they do not consume healthy foods as established in this study.

A systematic review assessed the relationship between socioeconomic status and NCDs and established that burden of behavioral risk factors is affected by socioeconomic position within low and middle-income countries (LLMICs) (33). The study established that alcohol use (not necessarily the harmful ones) and tobacco use were more prevalent among persons of low socioeconomic status. They also established that higher socioeconomic status groups tended to have higher levels of physical inactivity and more fats, salt, and processed foods than low socioeconomic groups putting them at a higher risk of NCDs (33).

This study has further that less poor households spent significantly more money on buying foods from different food retailers compared to the poorest households. The less poor households spent more money on food from supermarkets, and specialized shops compared to the poorest households. Overall, the less poor households spent more money purchasing foods food compared to the poorest households (Table 5). Supermarkets and specialized shop marketing activities have a major influence on consumer food purchases. A recent study established that supermarket circulars in most of the countries examined include a high percentage of discretionary foods, and therefore promote unhealthy eating behaviors that contribute to the global obesity epidemic (34). A significant positive relationship was observed between socioeconomic status and the total amount spent on food by households (Table 7). Another study done in Canada found that expenditures on food were lower among low-income households compared with high-income households (35).

A previous study concluded that healthier dietary diversity patterns have been associated with high socioeconomic status in low and middle-income countries (36). A study by Morseth et al. (31) aimed to investigate the association between socioeconomic status and dietary quality. The study was done among adult men and women living in a refugee camp in Algeria, which is different from our study setting. The findings, therefore, may not be directly comparable to our study which was a household survey done among people having a normal life: nonetheless, the study indicated that people with better socioeconomic status had higher dietary diversity scores. The findings concur with our study's result which revealed that individuals from less poor households had better dietary diversity scores compared to individuals from the poorest households, and this concurs with the findings of other studies.

International policies have now focused on ways of the increasing availability of inexpensive, high-calorie foods from staple cereal crops, which has reduced hunger for many. However, this has also affected food diversity and has displaced local, often healthier, and diets (1). Access to diverse, micronutrient-rich foods such as fresh fruits, vegetables, legumes, pulses, and nuts has not improved equally for everyone, and unhealthy foods with salt, sugars, saturated fats, and trans fats have become cheaper and more widely available (2). There is evidence that supermarkets and specialized shops promote the marketing of unhealthy foods and this will require the national and county governments should ensure that supermarkets are restricted from promoting unhealthy food marketing to reduce the incidence of NCDs. Another study done in Nepal showed that wealth status was associated with not consuming vitamin A-rich vegetables but consumption was influenced by factors such as age and province of residence (37). Other studies' findings, however, are contrasting with our results. A study done in India indicated that food consumption was not related to the wealth status of the family but rather to the maternal level of education (11).

Inequalities in access and consumption of quality foods possess a risk to risk of non-communicable diseases such as obesity (38), and malnutrition in human diets (39). Furthermore, poor diet quality is linked to the development of diet-related disorders such as diabetes, cardiovascular disease, and stroke (17). In Kenya, a study assessed the relationship between food security, health, and wealth status. The results showed that the risk of stunting increased by 12% among children from food-insecure households and food security and wealth status jointly increased the risk of stunting (18). Many developing nations are working to identify underlying gaps of inequalities in the population that will help inform policy formulation, which will drive interventions and minimization of risks among the populations.

## Limitations of the study

The study acknowledges several limitations. Firstly, the data provided by the respondents on the amount spent on food in the households may not be accurate due to either recall bias, respondent's unwillingness to give the correct information or poor documentation. The difficulties of collecting income or consumption expenditure data for health research in low-income countries remain, and further alternatives to the wealth index approach are also limited (40). However, use of durable assets to establish socioeconomic status has been recommended over expenditure of income data due to the fact that durable assets remain stable for longer period compared to expenditure or income data (40).

Secondly, this analysis is based on cross-sectional data and is estimating food purchasing practices in a month and food consumption on the previous day which may not provide adequate data to assess the impact of the actions. A prospective cohort study may provide a better option to monitor expenditures especially based on expenditure diaries kept by households. Lastly, we excluded households whose heads were aged below 18 years due to ethical considerations. This may have caused a selection bias since our data may not be generalizable to the population of households' heads < 18 years old. We did not have data to show the potential impact of the selection bias in our study. It's still unclear the proportion of child-headed households in Kenya. National KDHS only estimated that 1.2% of children are not living with their parents (41). We presumed that expenditure patterns among household members who are adults may not be different from those whose heads are under 18 years hence the impact of this bias may be limited. Hence, we don't expect this number of child-headed households to affect our findings significantly.

## Conclusion

There still exists significant socioeconomic inequalities in food purchasing and expenditure patterns which may predispose individuals to unhealthy foods and risk falling more into food poverty. Individuals with lower higher SES have different consumption and expenditures on unhealthy foods and each SES group is exposed to the risk of NCDs. As the countries progress toward attaining universal health coverage and reducing the burden of non-communicable diseases, there is a need to design policies that ensure access to healthy foods for all populations based on their socioeconomic status while at the same time the poor individuals cushioned against overspending above their ability to access healthy foods.

## Data availability statement

The raw data supporting the conclusions of this article will be made available by the authors, without undue reservation.

## Ethics statement

This study was approved by the Scientific and Ethics Committee (SERU) of the Kenya Medication Research Institute reference KEMRI/SERU/CGHR/174/3730. The approval proposal included a detailed consent form for all study participants. The patients/participants provided their written informed consent to participate in this study.

## Author contributions

VW conceived and designed the analysis and analyzed the data. RM, MP, PW, CL, and CO coordinated and performed the study. VW, LF, PW, MP, RM, CL, EM, ET-M, and CO drafted the manuscript. All authors read and approved the final manuscript.
